# Supplementary material for: Defining the relationship between maternal care behavior and sensory development in Wistar rats: Auditory periphery development, eye opening and brain gene expression
Source: PLoS One. 2020 Aug 21;15(8):e0237933. doi: 10.1371/journal.pone.0237933 (PMC7442246; doi:10.1371/journal.pone.0237933)
Supplement: S1 Table — (DOCX) [file pone.0237933.s001.docx]

**S1 Table. Bregma/Lambda reference coordinates (in mm) used for dissection of brain regions in this study.**

| **Age** | **Cochlear Nucleus** | **Pons** | **Inferior Colliculus** | **Auditory Cortex** | **Visual Cortex** |
| --- | --- | --- | --- | --- | --- |
| **P0** | **-5.00/-1.60** | **-4.40/-1.00** | **-4.80/-1.40** | **-2.00/1.40** | **-2.40/1.00** |
| **P7** | **-7.60/-2.20** | **-6.80/-1.40** | **-7.40/-2.00** | **-4.6/0.80** | **-5.60/-0.20** |
| **P15** | **-8.00/-1.80** | **-7.40/-1.20** | **-7.40/-1.20** | **-4.60/1.60** | **-5.80/0.40** |
| **P21** | **-8.00/-0.40** | **-7.40/0.20** | **-7.20/0.40** | **-4.60/3.00** | **-6.40/1.20** |
