# Supplementary material for: Defining the relationship between maternal care behavior and sensory development in Wistar rats: Auditory periphery development, eye opening and brain gene expression
Source: PLoS One. 2020 Aug 21;15(8):e0237933. doi: 10.1371/journal.pone.0237933 (PMC7442246; doi:10.1371/journal.pone.0237933)
Supplement: S2 Table — (DOCX) [file pone.0237933.s002.docx]

**S2 Table. Fold-change in brain region expression of genes expressed in vascular, glial cells and neurons.**

| Gene | Region | P0 | LG | P7 | P15 | P21 | ANOVA |
| --- | --- | --- | --- | --- | --- | --- | --- |
| *Olig2* | CN | 1.0±0.3 | Low | 1.7±0.9 | 2.2±0.5 | **3.9±0.9** | F(6,14)=3.503, p=0.0250 |
|  |  |  | High | 2.5±1.5 | 2.7±0.9 | **3.9±0.7** |  |
|  | Pons | 1.0±0.1 | Low | 1.1±0.3 | 1.5±0.4 | 1.8±0.3 | F(6,14)=0.9427, p=0.4959 |
|  |  |  | High | 1.2±0.5 | 1.9±0.8 | 2.0±0.1 |  |
|  | IC | 1.0±0.2 | Low | 3.5±2.9 | 3.0±1.3 | 1.9±0.5 | F(6,14)=0.5380, p=0.7710 |
|  |  |  | High | 1.4±0.6 | 1.8±0.6 | 1.4±0.3 |  |
|  | ACX | 1.0±0.2 | Low | 1.0±0.8 | 1.4±0.6 | 1.5±0.1 | F(6,14)=0.9075, p=0.5170 |
|  |  |  | High | 1.8±0.4 | 1.0±0.2 | 2.0±0.2 |  |
|  | VCX | 1.0±0.3 | Low | 1.6±0.9 | 1.5±0.4 | 3.2±0.3 | F(6,14)=1.152, p=0.3840 |
|  |  |  | High | 1.9±0.9 | 1.8±0.8 | 2.7±0.6 |  |
| *Mbp* | CN | 1.0±0.3 | Low | 0.4±0.05 | 0.3±0.1 | **1.8±0.5** | F(6,14)=4.889, p=0.0068 |
|  |  |  | High | 0.7±0.2 | 0.6±0.1 | 1.4±0.1 |  |
|  | Pons | 1.0±0.1 | Low | 0.6±0.1 | **0.4±0.07** | 0.8±0.1 | F(6,14)=1.330, p=0.3076 |
|  |  |  | High | 0.7±0.1 | 0.7±0.4 | 0.1±0.07 |  |
|  | IC | 1.0±0.2 | Low | 0.7±0.2 | 1.1±0.6 | 2.1±0.5 | F(6,14)=4.810, p=0.0073 |
|  |  |  | High | 1.0±0.4 | 0.7±0.3 | **2.8±0.4** |  |
|  | ACX | 1.0±0.08 | Low | 1.1±0.3 | 2.2±0.6 | **5.3±0.2** | F(6,14)=10.17, p=0.0002 |
|  |  |  | High | 0.9±0.3 | 1.2±0.2 | **2.8±0.3** |  |
|  | VCX | 1.0±0.3 | Low | 0.4±0.2 | 1.1±0.3 | **3.5±0.06** | F(6,14)=17.01, p<0.0001 |
|  |  |  | High | 0.6±0.3 | 1.4±0.4 | **3.0±0.2** |  |
| *Aqp4* | CN | 1.0±0.4 | Low | 5.5±1.1 | **8.4±1.1** | **15.8±1.3** | F(6,14)=13.85, p<0.0001 |
|  |  |  | High | **4.0±1.4** | **9.0±2.5** | **13.6±1.2** |  |
|  | Pons | 1.0±0.2 | Low | 1.6±0.3 | **3.9±1.1** | **5.9±0.6** | F(6,14)=6.299, p=0.0022 |
|  |  |  | High | 1.8±0.4 | **3.6±1.6** | **6.1±0.2** |  |
|  | IC | 1.0±0.3 | Low | 4.3±1.6 | **12.4±4.6** | **18.5±3.5** | F(6,14)=7.511, p=0.0010 |
|  |  |  | High | 3.0±1.2 | **9.5±1.8** | **18.3±2.8** |  |
|  | ACX | 1.0±0.4 | Low | 2.6±0.5 | **9.5±3.9** | **15.7±0.3** | F(6,14)=13.80, p<0.0001 |
|  |  |  | High | 6.0±1.1 | **9.7±1.7** | **18.3±1.2** |  |
|  | VCX | 1.0±0.3 | Low | 2.2±1.0 | **7.1±2.2** | **13.8±1.0** | F(6,14)=18.11, p<0.0001 |
|  |  |  | High | 2.1±1.3 | **6.7±2.1** | **16.7±1.2** |  |
| *Gjd2* | CN | 1.0±0.3 | Low | 0.9±0.4 | 0.6±0.2 | 2.1±0.8 | F(6,14)=1.083, p=0.4181 |
|  |  |  | High | 1.6±0.9 | 1.1±0.2 | 2.0±0.7 |  |
|  | Pons | 1.0±0.2 | Low | 0.6±0.4 | 0.6±0.2 | 0.6±0.08 | F(6,14)=0.6750, p=0.6722 |
|  |  |  | High | 0.7±0.1 | 0.6±0.2 | 0.6±0.05 |  |
|  | IC | 1.0±0.1 | Low | 0.8±0.3 | 1.3±0.7 | 1.3±0.2 | F(6,14)=0.3500, p=0.8983 |
|  |  |  | High | 1.3±0.7 | 0.7±0.06 | 0.8±0.1 |  |
|  | ACX | 1.0±0.2 | Low | 2.4±0.7 | 2.3±0.1 | **3.3±0.2** | F(6,14)=3.715, p=0.0202 |
|  |  |  | High | **3.1±0.9** | **4.1±0.7** | **3.7±0.3** |  |
|  | VCX | 1.0±0.6 | Low | 0.6±0.3 | 0.9±0.2 | 1.1±0.1 | F(6,14)=0.2988, p=0.9273 |
|  |  |  | High | 0.8±0.4 | 1.1±0.2 | 1.0±0.1 |  |
| *Gja1* | CN | 1.0±0.6 | Low | 0.8±0.3 | 0.9±0.1 | **2.4±0.4** | F(6,14)=4.482, p=0.0098 |
|  |  |  | High | 0.9±0.3 | 1.3±0.3 | **2.6±0.3** |  |
|  | Pons | 1.0±0.4 | Low | 1.0±0.1 | 1.5±0.3 | **3.6±0.6** | F(6,14)=12.70, p<0.0001 |
|  |  |  | High | 1.5±0.4 | 1.1±0.2 | **3.6±0.1** |  |
|  | IC | 1.0±0.3 | Low | 1.7±1.5 | 2.0±0.7 | 2.9±0.8 | F(6,14)=3.069, p=0.0393 |
|  |  |  | High | 0.9±0.3 | 1.0±0.3 | **4.3±0.2** |  |
|  | ACX | 1.0±0.2 | Low | 1.6±0.4 | **5.8±1.6** | **13.6±0.8** | F(6,14)=42.86, p<0.0001 |
|  |  |  | High | 2.6±0.4 | **8.4±0.8** | **13.9±0.7** |  |
|  | VCX | 1.0±0.3 | Low | 1.7±0.7 | 6.8±0.8 | **10.1±5.1** | F(6,14)=5.387, p=0.0045 |
|  |  |  | High | 1.7±1.0 | 5.5±1.3 | **14.4±1.6** |  |
| *Kcna3* | CN | 1.0±0.4 | Low | 0.7±0.2 | 0.7±0.2 | 1.2±0.3 | F(6,14)=1.436, p=0.2693 |
|  |  |  | High | 1.3±0.4 | 0.9±0.2 | 1.8±0.4 |  |
|  | Pons | 1.0±0.5 | Low | 0.5±0.3 | 0.7±0.2 | 0.5±0.2 | F(6,14)=0.7022, p=0.6529 |
|  |  |  | High | 0.6±0.04 | 0.4±0.01 | 0.7±0.09 |  |
|  | IC | 1.0±0.06 | Low | 1.0±0.5 | 2.7±1.2 | 1.4±0.4 | F(6,14)=1.477, p=0.2559 |
|  |  |  | High | 1.3±0.6 | 0.9±0.3 | 2.3±0.3 |  |
|  | ACX | 1.0±0.09 | Low | 1.1±0.5 | 1.4±0.6 | 1.8±0.2 | F(6,14)=2.124, p=0.1153 |
|  |  |  | High | 1.3±0.4 | 1.3±0.01 | **2.5±0.2** |  |
|  | VCX | 1.0±0.6 | Low | 0.3±0.2 | 0.4±0.1 | 0.6±0.02 | F(6,14)=1.225, p=3509 |
|  |  |  | High | 0.3±0.2 | 0.4±0.1 | 0.7±0.02 |  |
| *Panx2* | CN | 1.0±0.8 | Low | 0.4±0.3 | 0.7±0.2 | 0.8±0.07 | F(6,14)=0.4737, p=0.8168 |
|  |  |  | High | 0.6±0.4 | 1.0±0.4 | 1.2±0.08 |  |
|  | Pons | 1.0±0.4 | Low | 1.2±0.6 | 1.8±0.4 | 2.0±0.3 | F(6,14)=0.7821, p=0.5977 |
|  |  |  | High | 1.7±0.6 | 2.9±1.7 | 2.3±0.05 |  |
|  | IC | 1.0±0.4 | Low | 0.9±0.8 | 2.3±1.0 | 2.3±0.7 | F(6,14)=1.243, p=0.3431 |
|  |  |  | High | 1.7±0.8 | 1.1±1.0 | 2.7±0.7 |  |
|  | ACX | 1.0±0.7 | Low | 0.9±0.3 | 2.4±0.9 | **2.6±0.4** | F(6,14)=4.224, p=0.0124 |
|  |  |  | High | 0.8±0.3 | **2.7±0.1** | **3.2±0.2** |  |
|  | VCX | 1.0±0.5 | Low | 1.1±0.8 | **3.7±0.9** | **4.4±0.7** | F(6,14)=3.780, p=0.0189 |
|  |  |  | High | 1.4±0.8 | **3.3±1.0** | **4.0±0.5** |  |
| *Slc17a8* | CN | 1.0±0.9 | Low | 0.1±0.04 | 0.05±0.01 | 0.05±0.02 | F(6,14)=0.9575, p=4894 |
|  |  |  | High | 0.8±0.6 | 0.05±0.01 | 0.06±0.04 |  |
|  | Pons | 1.0±0.5 | Low | 0.7±0.3 | **0.1±0.09** | 0.3±0.2 | F(6,14)=1.936, p=0.1447 |
|  |  |  | High | 0.4±0.1 | **0.07±0.04** | 0.3±0.05 |  |
|  | IC | 1.0±0.5 | Low | 1.6±1.2 | 0.5±0.3 | 0.5±0.2 | F(6,14)=0.9556, p=0.4882 |
|  |  |  | High | 1.2±0.2 | 0.2±0.05 | 0.6±0.3 |  |
|  | ACX | 1.0±0.2 | Low | 5.0±1.0 | 6.0±4.0 | 11.0±7.0 | F(6,14)=1.015, p=0.4543 |
|  |  |  | High | 10.0±3.0 | 6.0±2.0 | 6.0±0.4 |  |
|  | VCX | 1.0±0.9 | Low | 0.3±0.1 | 0.3±0.1 | 0.3±0.08 | F(6,14)=0.5653, p=0.7512 |
|  |  |  | High | 0.2±0.04 | 0.4±0.1 | 0.4±0.1 |  |
