# Supplementary material for: Defining the relationship between maternal care behavior and sensory development in Wistar rats: Auditory periphery development, eye opening and brain gene expression
Source: PLoS One. 2020 Aug 21;15(8):e0237933. doi: 10.1371/journal.pone.0237933 (PMC7442246; doi:10.1371/journal.pone.0237933)
Supplement: S3 Table — (DOCX) [file pone.0237933.s003.docx]

**S3 Table. Genes that did not show changes in this study.**

| Gene | Region | P0 | LG | P7 | P15 | P21 | ANOVA |
| --- | --- | --- | --- | --- | --- | --- | --- |
| *Otx2* | CN | 1.0±0.8 | Low | 0.1±0.03 | 0.1±0.02 | 0.5±0.07 | F(6,14)=1.106, p=0.4064 |
|  |  |  | High | 0.2±0.1 | 0.2±0.06 | 0.3±0.1 |  |
|  | Pons | 1.0±0.2 | Low | 1.1±0.9 | 1.0±0.6 | 0.8±0.2 | F(6,14)=0.5198, p=0.7841 |
|  |  |  | High | 1.6±0.5 | 1.0±0.8 | 0.3±0.05 |  |
|  | IC | 1.0±0.5 | Low | 0.3±0.06 | 1.4±0.8 | 0.4±0.1 | F(6,14)=1.060, p=0.4302 |
|  |  |  | High | 0.4±0.2 | 0.5±0.1 | 0.4±0.3 |  |
|  | ACX | 1.0±0.5 | Low | 0.3±0.3 | 0.3±0.1 | 1.3±0.05 | F(6,14)=1.644, p=0.2075 |
|  |  |  | High | 2.0±1.3 | 0.3±0.1 | 1.3±0.3 |  |
|  | VCX | 1.0±0.07 | Low | 0.8±0.6 | 0.1±0.04 | 0.6±0.2 | F(6,14)=0.9685, p=0.4808 |
|  |  |  | High | 0.2±0.1 | 0.05±0.02 | 0.7±0.2 |  |
| *Wnt7b* | CN | 1.0±0.1 | Low | 0.6±0.3 | 0.6±0.1 | 1.3±0.2 | F(6,14)=1.969, p=0.1389 |
|  |  |  | High | 0.9±0.3 | 1.2±0.1 | 1.4±0.2 |  |
|  | Pons | 1.0±0.06 | Low | 0.7±0.1 | 1.3±0.3 | 1.4±0.2 | F(6,14)=1.592, p=0.2215 |
|  |  |  | High | 1.2±0.2 | 1.1±0.4 | 1.6±0.1 |  |
|  | IC | 1.0±0.4 | Low | 0.5±0.3 | 1.3±0.5 | 1.2±0.4 | F(6,14)=1.672, p=0.2004 |
|  |  |  | High | 0.7±0.2 | 0.5±0.2 | 1.6±0.3 |  |
|  | ACX | 1.0±0.2 | Low | 0.4±0.1 | 0.7±0.4 | 0.9±0.04 | F(6,14)=1.009, p=0.4577 |
|  |  |  | High | 0.6±0.2 | 0.8±0.3 | 1.0±0.1 |  |
|  | VCX | 1.0±0.2 | Low | 0.5±0.3 | 0.9±0.2 | 1.5±0.2 | F(6,14)=0.7773, p=6010 |
|  |  |  | High | 1.3±1.0 | 1.0±0.4 | 1.6±0.04 |  |
| *Gja4* | CN | 1.0±0.4 | Low | 1.6±1.4 | 0.2±0.04 | 2.1±1.0 | F(6,14)=3.523, p=0.0245 |
|  |  |  | High | 4.3±3.7 | 0.3±0.03 | 1.7±0.2 |  |
|  | Pons | 1.0±0.3 | Low | 1.5±1.1 | 0.3±0.08 | 0.9±0.3 | F(6,14)=1.342, p=0.3028 |
|  |  |  | High | 2.7±1.4 | 0.4±0.3 | 0.7±0.1 |  |
|  | IC | 1.0±0.09 | Low | 14.9±14.7 | 0.5±0.2 | 2.1±0.8 | F(6,14)=0.8967, p=0.5237 |
|  |  |  | High | 1.4±0.9 | 0.4±0.1 | 0.8±0.07 |  |
|  | ACX | 1.0±0.1 | Low | 1.5±0.7 | 1.3±0.1 | 2.9±0.8 | F(6,14)=0.7860, p=0.5951 |
|  |  |  | High | 1.6±0.5 | 1.6±0.1 | 2.4±0.07 |  |
|  | VCX | 1.0±0.3 | Low | 2.4±2.1 | 0.8±0.2 | 1.6±0.6 | F(6,14)=0.4685, p=0.8205 |
|  |  |  | High | 1.7±0.8 | 0.8±0.1 | 1.7±0.3 |  |
| *Gja5* | CN | 1.0±0.9 | Low | 0.4±0.4 | 0.03±0.0 | 2.2±0.8 | F(6,14)=1.968, p=0.1392 |
|  |  |  | High | 1.7±1.5 | 0.1±0.04 | 2.9±0.9 |  |
|  | Pons | 1.0±0.5 | Low | 0.3±0.2 | 0.1±0.1 | 0.3±0.1 | F(6,14)=1.038, p=0.4419 |
|  |  |  | High | 0.6±0.4 | 0.2±0.1 | 0.4±0.2 |  |
|  | IC | 1.0±0.3 | Low | 1.1±1.1 | 0.02±0.01 | 2.2±1.7 | F(6,14)=1.054, p=0.4332 |
|  |  |  | High | 0.2±0.1 | 0.06±0.05 | 0.4±0.1 |  |
|  | ACX | 1.0±0.3 | Low | 0.4±0.3 | 0.3±0.2 | 2.2±0.7 | F(6,14)=1.818, p=0.1673 |
|  |  |  | High | 2.5±1.7 | 0.1±0.04 | 1.9±0.7 |  |
|  | VCX | 1.0±0.8 | Low | 0.2±0.2 | 0.08±0.02 | 3.1±0.8 | F(6,14)=3.458, p=0.0261 |
|  |  |  | High | 2.0±1.5 | 0.08±0.02 | 4.4±1.5 |  |
| *Ano1* | CN | 1.0±0.5 | Low | 0.9±0.8 | 0.03±0.01 | 1.9±0.8 | F(6,14)=4.345, p=0.0111 |
|  |  |  | High | 2.3±1.9 | 0.09±0.02 | 1.6±0.2 |  |
|  | Pons | 1.0±0.5 | Low | 0.07±0.02 | 0.1±0.1 | 0.5±0.2 | F(6,14)=0.9298, p=0.5035 |
|  |  |  | High | 1.4±0.8 | 0.2±0.1 | 0.4±0.1 |  |
|  | IC | 1.0±0.2 | Low | 3.8±3.7 | 0.06±0.02 | 1.9±1.1 | F(6,14)=0.6851, p=0.6650 |
|  |  |  | High | 0.3±0.2 | 0.1±0.07 | 0.8±0.07 |  |
|  | ACX | 1.0±0.6 | Low | 3.1±3.0 | 0.2±0.08 | 2.1±0.8 | F(6,14)=1.002, p=0.4616 |
|  |  |  | High | 5.3±3.6 | 0.2±0.03 | 1.5±0.5 |  |
|  | VCX | 1.0±0.6 | Low | 1.8±1.8 | 0.1±0.02 | 2.7±1.6 | F(6,14)=1.216, p=0.3545 |
|  |  |  | High | 3.8±2.0 | 0.1±0.03 | 2.1±0.5 |  |
| *Panx1* | CN | 1.0±0.5 | Low | 1.2±0.7 | 0.3±0.09 | 1.4±0.4 | F(6,14)=3.556, p=0.0.0237 |
|  |  |  | High | 3.2±2.5 | 0.5±0.05 | 1.6±0.1 |  |
|  | Pons | 1.0±0.2 | Low | 0.9±0.6 | 0.2±0.07 | 0.6±0.2 | F(6,14)=1.811, p=0.1686 |
|  |  |  | High | 1.4±0.5 | 0.3±0.1 | 0.4±0.1 |  |
|  | IC | 1.0±0.2 | Low | 3.3±2.9 | 0.6±0.2 | 1.4±0.7 | F(6,14)=0.7003, p=0.0.6542 |
|  |  |  | High | 1.0±0.2 | 0.5±0.1 | 0.9±0.09 |  |
|  | ACX | 1.0±0.01 | Low | 1.0±0.4 | 0.9±0.5 | 1.2±0.07 | F(6,14)=0.4892, p=0.8059 |
|  |  |  | High | 1.3±0.5 | 0.6±0.1 | 1.1±0.06 |  |
|  | VCX | 1.0±0.3 | Low | 1.0±0.5 | 0.5±0.1 | 1.5±0.2 | F(6,14)=0.9549, p=4887 |
|  |  |  | High | 1.2±0.5 | 0.8±0.06 | 1.0±0.06 |  |
