# Supplementary material for: Defining the relationship between maternal care behavior and sensory development in Wistar rats: Auditory periphery development, eye opening and brain gene expression
Source: PLoS One. 2020 Aug 21;15(8):e0237933. doi: 10.1371/journal.pone.0237933 (PMC7442246; doi:10.1371/journal.pone.0237933)
Supplement: Attachment — Submitted filename: Response to Reviewers.docx [file pone.0237933.s009.docx]

PONE-D-20-07005
Robust range of auditory periphery development, eye opening, and brain gene expression in Wistar rat pups that experience variation in maternal backgrounds.
PLOS ONE

Dear Dr. Biagini,

We would like to thank the reviewers for their constructive reviews and comments. To comply with journal requirements we have updated the ethics statement in the Methods section as follows: ‘The Institutional Animal Care and Use Committee of the City College of New York specifically reviewed and approved this study.’ We have created 2 supplementary files with Tables. We have updated the Data Availability statement appropriately and provide excel files with the data used to make the figures and tables of the manuscript. Next is our point by point response to the comments made by two reviewers:

[Note: HTML markup is below. Please do not edit.]

Reviewers' comments:

Reviewer's Responses to Questions

**Comments to the Author**

1. Is the manuscript technically sound, and do the data support the conclusions?

The manuscript must describe a technically sound piece of scientific research with data that supports the conclusions. Experiments must have been conducted rigorously, with appropriate controls, replication, and sample sizes. The conclusions must be drawn appropriately based on the data presented.

Reviewer #1: Partly

Reviewer #2: Partly

2. Has the statistical analysis been performed appropriately and rigorously?

Reviewer #1: Yes

Reviewer #2: Yes

3. Have the authors made all data underlying the findings in their manuscript fully available?

The [PLOS Data policy](http://www.plosone.org/static/policies.action#sharing) requires authors to make all data underlying the findings described in their manuscript fully available without restriction, with rare exception (please refer to the Data Availability Statement in the manuscript PDF file). The data should be provided as part of the manuscript or its supporting information, or deposited to a public repository. For example, in addition to summary statistics, the data points behind means, medians and variance measures should be available. If there are restrictions on publicly sharing data—e.g. participant privacy or use of data from a third party—those must be specified.

Reviewer #1: Yes

Reviewer #2: Yes

4. Is the manuscript presented in an intelligible fashion and written in standard English?

PLOS ONE does not copyedit accepted manuscripts, so the language in submitted articles must be clear, correct, and unambiguous. Any typographical or grammatical errors should be corrected at revision, so please note any specific errors here.

Reviewer #1: Yes

Reviewer #2: Yes

5. Review Comments to the Author

Reviewer #1: This manuscript details effects of maternal licking and grooming behavior on the neurodevelopment of pups. The authors have mainly focused on the effects of maternal behavior on the development of auditory and visual cortices and respective outcomes. The wide range of testing paradigm was used such as analysis of ABR, micro-CT, time onset of eye opening and gene expression analysis of various pathways involved in growth, and related pathways. The authors found that maternal behavior does not significantly impact ABR onset. The observations indicated a delay between hearing onset and eye opening. The study provides novel indication about relationship between growth factor bdnf RNA expression and hypoxia-related pathway in the LG group.

Comments to the authors:
The study is very elaborate and robust methodology was followed. However, I have a few specific comments
Methods:

1. Page 5 line 109 to 121: The description of animals and the format of testing with numbers followed is elusive, it would be great if all those numbers that were used could be presented in a table format.

Authors response: Thank you for your suggestion. We revised and summarized adult animal information in Table 1 and pup information in Table 2. We edited the text on the first paragraph of “Experimental Design and Statistical Analysis” to account for this change.

2. Page 5 line 113: It is not mentioned where did these 160 females come from?

Authors response: This information is now found in Table 1.

3. Page 5 line 25 : The description of text does not match that of figures

Authors response: Thank you for pointing this out. We have revised the manuscript so that text matches the figures.

4. Page 7 line 164: what is the rationale for using median as a statistical entity here

Authors response: The coefficient k value data did not pass a normality test, so we used a non-parametric test to assess statistical differences between the medians of the data samples. We have updated the text in “Experimental Design and Statistical Analysis” to indicate when means or medians were used in statistical tests.

5. Page 9 line 211: Were the observations made manually or using any behavioral software, if manually were they done by the same individual or different? Because the observations can vary with individual perspectives, how was the variation controlled for?

Authors response: Thank you for your questions. We did not perform software analysis of behavioral video recordings. Instead, lead authors JQ and PS were trained to manually score maternal behaviors by senior author FC. Given the overwhelmingly large number of observations to complete in this study, we decided to recruit different groups of undergraduate students who helped us score maternal behavior. Motivated undergraduate student helpers were recruited by AR-C and were trained by JQ and PS. To improve training and assess scoring variation amongst student helpers, JQ and PS chaperoned each group of students for their first set of two or three observations. We estimate that variation across observers was smaller than 5%. For clarity, we have added text that indicates observations were performed by trained observers.

6. Page 11 line 273: What are the indications of looking for these genes, please provide some background while introducing these?

Authors response: Thank you for your suggestion. This is a point that overlaps with the comments of reviewer 2 below. We have made three changes to the manuscript. We rewrote the introduction to include a rationale for including the hypoxia-sensitive pathway and neurotrophin signaling in the gene expression screen. We provide more background in the Gene Expression section of the Methods to reference previous studies that identified the genes of interest. Lastly, we reorganized the presentation of gene expression data in two new tables (Table 4 and Table 5), and added two supplementary tables for data that is less relevant to the main focus of the paper (S1 Table and S2 Table).

7. Why were the litters selected based on summer and spring, what is the significance of seasons, what is the rationale behind this?

Authors response: Thank you for your question. The main reason to conduct observations in the spring and summer was the availability of space at the animal facility. We did not intend to study seasonal effects in this study, so we have removed any reference to seasons from the manuscript. As a result, we edited Panel A in Figure 2, eliminated panel D in Figure 2, an updated the text of the legend to Figure 3 and throughout the manuscript.

8. Did the authors take into consideration the effect of isoflurane on latency to onset of ABR?

Authors response: Thank you for your observation. We consider that anesthesia could be a confounding factor since it has suppressive effects on neural activity. We added a statement and references to support it in the discussion.

Figures and results and discussion

1. Page 5 line 125 : The figure 1D is not present, which figure are you referring to?

Authors response: Thank you for pointing this out. We have revised the text to match the figures throughout the manuscript.

2. Why are the figure legends for figure 7, 8 appearing before figure 4,5,6 ? I think they should be placed well in sequence or the text should be adjusted accordingly.

Authors response: Thank you for your suggestion. We have revised the text so that all the figure legends appear in the appropriate numerical sequence.

3. Page 16 line 372: Which figure are you talking about here, please mention in the text

Authors response: We did not generate a figure for the correlation analysis. After revising the manuscript we do not consider this analysis to be essential for the evaluation of the data. In the interest of integrating and shortening the manuscript (as suggested by reviewer 2) we removed this paragraph from the manuscript

4. How do the findings in the gene expression analysis correlate and reflect upon the ABR, eye opening or other changes in the pups of High and low LG dams, what are author’s insight about the changes at transcription level? Please include in discussion.

Authors response: We re-organized the discussion section to provide an interpretation of the gene expression data.

5. Is it possible to represent the results of gene expression analysis in tabular form and highlighting the significant ones, it will make the manuscript for comprehensive?

Authors response: Thank you for the suggestion. We replaced Figures 7, 8 and 9 with tables 4 and 5 which focus on the hypoxia-sensitive pathway and neurotrophin signaling, and genes related to these pathways. We generated two supplementary tables with results that are less relevant to the main focus of the paper.

6. Page 37 line 897: When the authors mention about this correlation, are they sure of a direct correlation or there are confounders due to variability in measurements by individuals?

Authors response: Thank you for pointing this out. It is entirely possible that due to variation of observations by human observers we did not find a correlation between these variables. As indicated in our reply to point 3, we have removed this paragraph from the manuscript.

General comments:
The manuscript is thoroughly written and grammatically sound, I just have a few general comments
1. About the title, would you be more specific when you say, ‘maternal background’, specifying that it is the behavioral background you are focusing on rather than the genetic?

Authors response: Thank you for your suggestion. We rewrote the Title to better reflect the aims and results of the study, and to indicate we used behavioral background as you suggest. As appropriate, we have indicated throughout the manuscript that we refer to LG as a maternal behavior and not a genetic trait.

2. Moving a lot of gene analysis data into tabular format will help decrease confusion to the reader.

Authors response: Thank you for your suggestion. We have simplified gene expression data by focusing our attention in fewer genes listed in Tables 4 and 5, and by creating two supplementary tables.

3. Please check and match figures to the figure legends.

Authors response: Thank you for your suggestion. We checked that figure legends match the content of the corresponding figures.

Reviewer #2: Review on PONE-D-20-07005:

The authors investigated the effect of maternal licking and grooming (LG) during the first week of life on the timing of hearing onset in Wistar rat pups. In this regard, they examined some parameters such as auditory brainstem responses (ABR), tracking of eye opening (EO), micro-CT X-ray tomography, and qRT-PCR to monitor neurodevelopmental changes in the female and male offspring exposed to different maternal LG. The authors found no significant effect of LG on the mentioned parameters but found that the hypoxia-sensitive pathway is regulated in subcortical and cortical auditory brain regions before hearing onset, and also the role of maternal LG in regulation of BDNF signaling in auditory cortex after hearing onset. However some major points have not been properly addressed as follows:

Title: The title is not in accordance with the findings of the study.

Abstract: Lines 32-34 of this section, are not correlated with the main findings of the research.

Authors response: Thank you for pointing this out. We have removed the referred text from the abstract. The abstract has been edited to reflect the main goals and findings of the study.

Introduction: According to the main findings of the research it is suggested to re-write the introduction in order to get the reader aware of what is presented in the following parts of the manuscript. For example it is needed to be written something about hypoxia-sensitive pathway and BDNF signaling in relation to hearing onset. Also, the importance of the research and the possible application and benefit of the obtained results should be mentioned.

Authors response: Thank you for making this suggestion. We have re-written the second and third paragraphs of the introduction to highlight the relevance of the research on hypoxia-sensitive and neurotrophin pathways during auditory system development, including statements of significance and possible clinical applications of the research.

Discussion: As mentioned above in the comments for introduction, the first paragraph of the discussion also needs revision. The second and third parts of the discussion is better to be presented before the first part.

Authors response: We have re-organized the discussion section as suggested.

-The conclusion needs revision, it should contain a take-home message actually according to the main findings of the study, not again discussing on other studies.
-As a whole, the manuscript should be integrated and shortened.

 Authors response: We have revised the manuscript trying to integrate and shorten the text. We have tried to focus our discussion on the main findings of the study and less on results from other studies.

6. PLOS authors have the option to publish the peer review history of their article ([what does this mean?](https://journals.plos.org/plosone/s/editorial-and-peer-review-process#loc-peer-review-history)). If published, this will include your full peer review and any attached files.


If you choose “no”, your identity will remain anonymous but your review may still be made public.


**Do you want your identity to be public for this peer review?** For information about this choice, including consent withdrawal, please see our [Privacy Policy](https://www.plos.org/privacy-policy).

Reviewer #1: No

Reviewer #2: No


[NOTE: If reviewer comments were submitted as an attachment file, they will be attached to this email and accessible via the submission site. Please log into your account, locate the manuscript record, and check for the action link "View Attachments". If this link does not appear, there are no attachment files to be viewed.]


While revising your submission, please upload your figure files to the Preflight Analysis and Conversion Engine (PACE) digital diagnostic tool, <https://pacev2.apexcovantage.com/>. PACE helps ensure that figures meet PLOS requirements. To use PACE, you must first register as a user. Registration is free. Then, login and navigate to the UPLOAD tab, where you will find detailed instructions on how to use the tool. If you encounter any issues or have any questions when using PACE, please email us at [figures@plos.org](mailto:figures@plos.org). Please note that Supporting Information files do not need this step.

*In compliance with data protection regulations, you may request that we remove your personal registration details at any time.* [*(Remove my information/details)*](https://www.editorialmanager.com/pone/login.asp?a=r)*. Please contact the publication office if you have any questions.*
