# Supplementary material for: Defining the relationship between maternal care behavior and sensory development in Wistar rats: Auditory periphery development, eye opening and brain gene expression
Source: PLoS One. 2020 Aug 21;15(8):e0237933. doi: 10.1371/journal.pone.0237933 (PMC7442246; doi:10.1371/journal.pone.0237933)
Supplement: Attachment — Submitted filename: Response to Reviewers.docx [file pone.0237933.s010.docx]

PONE-D-20-07005R1
PONE-D-20-07005
Defining the relationship between maternal care behavior and sensory development in Wistar rats: auditory periphery development, eye opening and brain gene expression
PLOS ONE

6. Review Comments to the Author

Please use the space provided to explain your answers to the questions above. You may also include additional comments for the author, including concerns about dual publication, research ethics, or publication ethics. (Please upload your review as an attachment if it exceeds 20,000 characters)

Reviewer #1: The authors have addressed all the major concerns. And the manuscript is acceptable in its current form. However, I have a few minor comments as follows:
1. Page 3 line 62: Is there a proof of maternal LG behavior leading to hypoxia or oxygen deficiency in pups, please mention background, mention studies (if any) indicating the same.

Authors reply: Thank you for raising this point. We found two articles that are relevant to document a link between maternal LG behavior and oxygen deficiency in pups. We added the new text and cited the references on page 3 lines 63-66.

2. Figure 4 : It will be more clear, If the symbol legends can be place on the figure itself.

Authors reply: Thank you for the suggestion. We have added symbol legends in Figure 4.

3. Page 30 line 616: Please specify the group you are talking about.

Authors reply: Thank you for raising this issue. We have revised the text to clarify the LG groups on page 30 lines 623-628.

4. Page 32: Conclusion: I have the same concern, how are you justifying the relationship between maternal behavior and hypoxia in pups? Or you are concluding on the basis of transcription changes, if so please mention. Also mention the same while introducing hypoxia pathway in the introduction by citing relevant studies.

Authors reply: See our response to points 1 and 3 above. We have been careful in the discussion. For example, in page 30 lines 628 to 634 we state: ”Assuming that transcriptional down regulation of *Egln1* causes reduced levels of mRNA transcripts and Phd2 in cells of the auditory system, this result would suggest that cells in the auditory system of neonate rats may have a differential sensitivity to oxygen challenges during postnatal development. This interpretation is consistent with results that showed higher vulnerability of P7 compared to P1 rats when exposed to hypoxia-ischemia, and with studies that tracked long-term maternal behavioral, learning and auditory deficits in rats exposed to perinatal anoxia at birth [16, 48, 49].” To address your concern in the conclusion, we state more carefully (page 32 lines 680 and 681: “The results of a gene expression screen suggest that sensitivity to hypoxic challenge might be widespread in the auditory system of neonate rats before hearing onset” We updated the abstract accordingly on page 2 lines 45 and 46.

Reviewer #3: The authors examined the effect of maternal licking and grooming (LG) during the first week of life on the timing of hearing onset in Wistar rat pups by different approaches. Among them auditory brainstem responses (ABR), tracking of eye opening (EO), micro-CT X-ray tomography, and gene expression analysis in 5 different brain regions. The authors found statistically significant increases in the relative mRNA levels of four genes involved in neurotrophin signaling in auditory brain regions from pups of different LG backgrounds. Nevertheless, the authors' hypothesis that LG backgrounds affect the timing of ABR onset, EO, and the relative mRNA levels of genes involved in the hypoxia-sensitive pathway has not been confirmed.

This paper is well presented and has good potential. The topic of the manuscript is interesting because the disruption of infant-mother interaction (as seen in low-LG mothers and some animal stress models) results in multiple delayed negative effects on behavioral phenotype and cognitive performance.

Of note, that the number of such studies at several time points is limited. There are, however, some points that need attention.

1. The number of animals used in some analysis is small, at least 6 - 8 (from at least 4 different litters) animals should have been used.

Authors reply: Thank you for your comment. In ideal conditions we would have liked to process more samples in the gene screen experiments. To adapt our study to the constraints of our budget we decided for triplicate samples from pups of one, two or three litters as described in the methods.

2. Different regions were used in the study: cochlear nucleus, pons (ventral brainstem containing the acoustic stria), inferior colliculus, temporal cortex (here referred to as auditory cortex), and occipital cortex (here referred as visual cortex). Please describe in more detail how they were dissected (indicating the required coordinates according to the atlas of the brain).

Authors reply: Thank you for your comment. We have updated the Gene Expression Methods section as requested (page 10 lines 230-237) with a brief description of the landmarks used to dissect brainstem structures according to Rodriguez-Contreras et al. (2014), and the approximate bregma/lambda coordinates according to the most up to date atlas of the neonate rat brain (Khazipov et al. 2015) in S1 Table.

3. For PCR analysis, only one reference gene was used. Although this gene is often used as a housekeeping gene, have you checked that it is not differentially expressed in these brain regions?

Authors reply: Thank you for raising this point. We were careful to check the housekeeping gene across all ages and LG conditions, serving as an internal control for the PCR analysis. It is important to note that we decided to compare mRNA levels for each gene as a fold change with respect to P0. For the sake of transparency we included the fold change data for the housekeeping gene *Actb* in Table 4, and revised the text on page 22 lines 498-501.
